# Cannabis use in adolescence and young adulthood and its effects on brain structure and function: a scoping review

**DOI:** 10.3389/fpsyt.2025.1644105

**Published:** 2025-09-03

**Authors:** Lilith Nosko, Candice E. Crocker, Phil G. Tibbo

**Affiliations:** ^1^ Department of Psychiatry, Dalhousie University, Halifax, NS, Canada; ^2^ Research and Innovation, Nova Scotia Health Authority, Halifax, NS, Canada; ^3^ Department of Diagnostic Radiology, Dalhousie University, Halifax, NS, Canada

**Keywords:** cannabis, adolescence, young adulthood, brain, neuroimaging

## Abstract

**Introduction:**

Adolescence and young adulthood are simultaneously periods of significant brain development and the ages in which people often initiate cannabis use. This has led to significant interest in researching the effects that cannabis use in this period might have on the brains of users. This scoping review aims to summarize existing neuroimaging research on the effect of cannabis use in adolescence and/or young adulthood (ages 14-25) on brain structure, function, and metabolite concentrations.

**Methods:**

Following scoping review methodology, databases containing neuroimaging studies assessing the effects of cannabis use between the ages of 14 and 25 on brain structure, function, and metabolite concentrations were searched.

**Results:**

Our search yielded 3901 sources, of which 99 met inclusion criteria. The majority of included papers (84/99) found differences in the brain structure, function, and/or metabolite concentrations of adolescent/young adult cannabis users compared to non-using controls. Fewer studies explicitly assessed sex/gender differences, with 5 finding that sex/gender influenced the effect of cannabis use on the brain.

**Conclusion:**

Based on the findings of this review, there is considerable evidence to suggest that cannabis use in adolescence/young adulthood causes changes in the brains of users, however, the low quality of relevant research and scarcity of long term follow up studies, in addition to the heterogeneity of the existing research suggests that more work needs to be done to understand this relationship.

## 1 Introduction

Cannabis use is common in adolescence and young adulthood (AYA), a period which ranges from approximately 10 years old to the mid-twenties (1). School-based studies have reported, for example, that approximately 1 in 5 students used cannabis in the past year, and among these students, 1 in 10 endorsed daily cannabis use (2). Of concern is that the rates of cannabis use among youth appear to be increasing over time (3). Within a 2023 survey of Canadians’ cannabis use, it was found that 43% of people between 16–19 years old and 48% of people between 20–24 years old reported past year cannabis use, which was almost twice the amount of those over the age of 25 (4). High rates of cannabis use in AYA are also seen internationally. A 2022 survey of 12^th^ grade students in the United States found that 30.7% of respondents endorsed past-year cannabis use (5). Similarly, a European survey reported a past-year cannabis use prevalence of 18.6% among individuals aged 15 to 24 (6). The use of cannabis during AYA is of particular interest as this time frame is known to be significant for brain maturational processes underlying important adult cognitive functions (7), such as cognitive control (8), working memory (9), risk-taking (10), and reward (11). Past research on the effects of cannabis use during this life-stage has associated it with a number of possible negative outcomes, such as a variety of cognitive deficits (12), an increased likelihood of developing an anxiety disorder (13) or a depressive disorder (14), engaging in problematic substance use (15), and increased suicidality (14, 16). Cannabis use during AYA has also been associated with a greater likelihood of, and earlier onset of psychotic disorders (17), as well as worsened clinical outcomes for those young adults with current psychotic disorders (18). To understand the effect that cannabis use in AYA has on the brain, leading to changes in mental health and cognition, neuroimaging research has been applied.

Despite there being no consensus on the long-term effects of cannabis on the brain in AYA, there is a mixed body of research which suggests that such effects might exist. For example, previous research has reported that, compared to non-users, AYA cannabis users have abnormalities in brain morphology (19), resting-state brain activity (20), task-based brain activity (21), and metabolite concentrations (22). While fewer, some research has found no effect of AYA cannabis use on various brain measures (23). This body of potentially contradictory evidence, when examined more closely, has clearly differing definitions of “cannabis user,” small sample sizes, flawed study designs, and heterogenous imaging protocols severely limiting the usefulness of these findings. This makes it exceedingly difficult to meaningfully gauge the overall tenor of the results being published and as such, literature reviews are positioned to distill and synthesize these bodies of work to better inform this research area of interest.

Two recent and relevant reviews on AYA cannabis use and brain effects (24, 25) have been published. One of the reviews (24) was more a narrative review, with some lack of clarity in its methodology [and adherence to established guidelines; see (26)]. The next most recent publication (25), a systematic review (*n* = 90 studies), included sources derived from a search conducted in 2019, thus a more recent review which includes publications completed over the last 5 years, and with consideration of brain metabolite concentrations and more refined imaging protocols, is warranted.

To summarize existing knowledge regarding the effect that AYA cannabis use has or does not have on the structure, function, and metabolite concentrations of the user’s brain, we conducted a scoping review. Scoping reviews are designed to provide a broad overview of what is- and remains to be known in a research field, as opposed to a systematic review, which focuses on answering a very specific question (27). A scoping review methodology was also chosen for this reason and because they are most appropriate for situations in which the chosen literature is highly heterogeneous and/or in situations where resources are limited (28), both of which are true for the current review.

## 2 Method

This scoping review was conducted in accordance with the Joanna Briggs Institute (JBI) methodology for scoping reviews (29). The protocol for the current review was preregistered on Open Science Framework on May 23, 2024 (30, 31).

### 2.1 Inclusion criteria

This review included peer-reviewed studies published in English, theses/dissertations, and preprints using brain imaging and comparing cannabis users to non-users between the ages of 14 and 25 years old. This range was selected based on the WHO’s definition of AYA [10–24 years; (1)], the age ranges commonly used in research on AYA cannabis use, and the age by which puberty has typically started (32), indicating adolescence. Studies examining participants older than this age range were included if it was confirmed that participants regularly used cannabis between our age-range of interest. In addition, cannabis must have been the participants’ primary substance and its use by the participants must have been quantified. Participants were allowed to have preexisting psychiatric conditions (except substance use disorders related to a substance other than cannabis). Sex and gender effects were collected for this review when possible.

### 2.2 Exclusion criteria

This review did not include papers which assessed the acute effects of cannabinoid administration on the brain or animal studies.

### 2.3 Types of sources

This scoping review considered both experimental and quasi-experimental study designs including randomized controlled trials, non-randomized controlled trials, before and after observational studies and interrupted time-series studies. In addition, analytical observational studies including prospective and retrospective cohort studies, case-control studies and analytical cross-sectional studies were considered for inclusion. Also considered were descriptive observational study designs. Systematic reviews that meet the inclusion criteria were included with any relevant sources assessed and extracted.

### 2.4 Search strategy

A three-step search strategy was utilized in this review, with no limit on date of publication. First, an initial limited search of Ovid MEDLINE (NLM, Wolters Kluwer), CINAHL (EBSCO), PsycINFO (American Psychological Association), and Embase (Elsevier) was undertaken to identify relevant articles on the topic. The key words contained in the titles and abstracts of relevant articles, and the subheadings used to describe the articles were then used to develop a full search strategy for each database outlined in **Appendix I**. Using these terms, searches were conducted in all the listed databases as well as Google Scholar and MedRxiv.

### 2.5 Study selection

**Figure 1:**
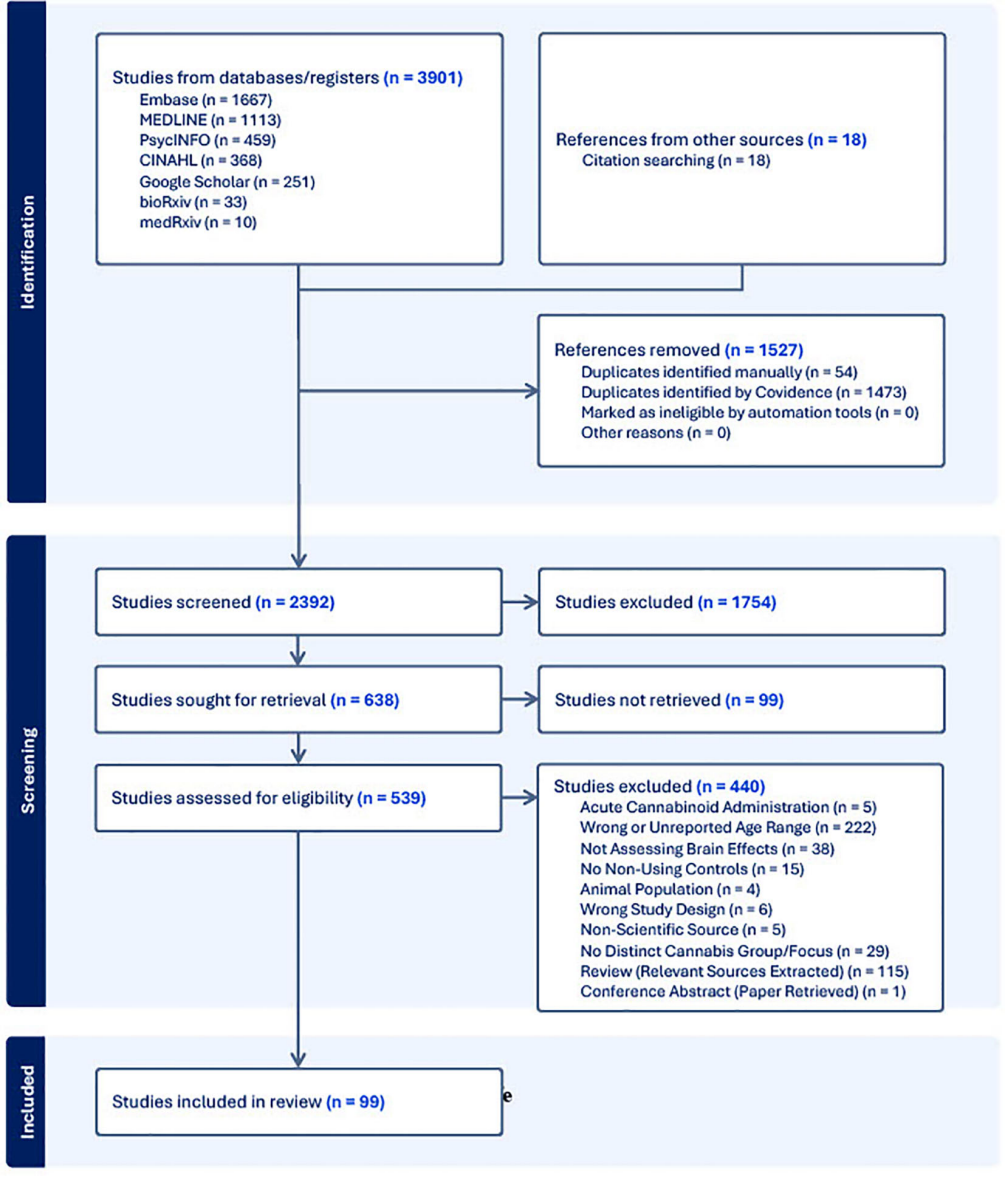
PRISMA-ScR diagram of the screening and extraction process for the current review.

Following the search of each database, all identified citations were collected and uploaded into Covidence (33) where duplicates were removed. Following a pilot test, titles and abstracts were screened by two independent reviewers (L.N., C.H.L.). Potentially relevant sources were then retrieved in full and subsequently assessed in detail against the inclusion criteria. The reference list of all included sources were screened for additional relevant studies. Reasons for exclusion of sources were recorded and reported in the Preferred Reporting Items for Systematic reviews and Meta-Analyses extension for Scoping Reviews [PRISMA-ScR; (34)] flow diagram (**Figure 1**). Any disagreements that arose between the reviewers at each stage of the selection process was resolved either through consensus, or if that was not possible, then with input from an additional reviewer (C.E.C., P.G.T.).

### 2.6 Data extraction

Data were extracted by two independent reviewers using a data extraction tool developed by the study team and completed in Covidence. From each paper the following was extracted: authors, year of publication, study design, sample size, mean age and age range of sample (divided into cannabis users and non-users), and relevant findings. Any disagreements that arose between the reviewers were resolved through discussion, or with input from an additional reviewer.

### 2.7 Sex and gender considerations

As a secondary objective, we aimed to determine if and how sex and/or gender influences the effect of AYA cannabis use on the brain. The World Health Organization’s definitions of sex and gender were used, that is, sex being an individual’s sex assigned at birth (i.e., male or female), while gender referred to an individual’s identity and societal role (i.e., man or woman) (35)). Included papers were categorized as having assessed sex/gender differences or not, with relevant sex/gender data extracted where available. Many of the studies that stated they assessed sex/gender were not explicit on the constructs they were assessing, in these cases we categorized them based on the language used (i.e., sex or gender).

## 3 Results

### 3.1 Characteristics of included studies

Our search yielded 99 papers that met our inclusion criteria (See **Figure 1**; a number of papers included multiple imaging modalities). Out of these, 46 utilized structural neuroimaging protocols and 56 functional neuroimaging protocols. Out of the structural imaging studies, 29 used structural magnetic resonance imaging (sMRI), 12 used diffusion tensor imaging (DTI), 4 used magnetic resonance spectroscopy (MRS), and 1 used another imaging modality (CT) (See **Table 1**). Of the functional imaging studies, 39 used task-based functional magnetic resonance imaging (fMRI), 10 used resting-state fMRI, 5 used electroencephalography (EEG), and 2 used positron emission tomography (PET) (see **Table 2**).

**Table 1:** Proportion of different structural imaging modalities utilized in studies included in the review.

Structural Imaging Modality	n (%)
DTI	12 (26.1)
sMRI	29 (63.0)
MRS	4 (8.7)
Other	1 (2.2)
Total	46

**Table 2:** Proportion of different functional imaging modalities utilized in studies included in the review.

Functional Imaging Modality	n (%)
Task-Based fMRI	39 (69.6)
Resting-State fMRI	10 (17.9)
EEG	5 (8.9)
PET	2 (3.6)
Total	56

Approximately 85% (84/99) of the included studies found at least one significant difference in various aspects of brain structure/function between individuals who used cannabis between the ages of 14 and 25 and those who did not. While all studies reported the gender and/or sex of the participants, only 17% (17/99) assessed sex/gender differences related to their reported outcome(s). Five (~5%) of these studies found a significant sex/gender difference. The aim of the remainder of this review is to discuss the results of the included papers (see **Tables 3**–**10** for summaries of all included studies).

**Table 3:** Summary of DTI studies.

Study	Study Design	Sample Size	Age of Users mean (SD), range	Age of non-users mean (SD), range	Changes associated with cannabis use*	Sex/Gender Differences*
(64)	Cross sectional study	14 users, 14 non-users.	19.3 (0.8), 18-21.2	18.5 (1.4), 17.3-21.5	•Reduced FA in the left posterior internal capsule/thalamic radiation, left middle temporal gyrus, right posterior internal capsule, right superior temporal gyrus, and right arcuate. •Reduced axial diffusivity in motor tracts. •Greater radial diffusivity and trace values in bilateral arcuate. Reduced FA only in the right arcuate.	•Not assessed.
(23)	Cross sectional study	25 users, 25 non-users.	20.40 (1.94), 17-23	19.76 (1.90), 17-23	•None reported.	•Not assessed.
(65)	Longitudinal study	23 users, 23 non-users.	BL: 19.45 (0.66), FU: 21.31 (2.43)	BL: 19.19 (2.31), FU: 21.79 (0.82)	•Attenuated increase of FA in left superior longitudinal fasciculus, left superior frontal gyrus, the left corticospinal tract, and the right anterior thalamic radiation. •Attenuated decrease of RD in right central and posterior superior longitudinal fasciculus, posterior cingulum, and corticospinal tract.	•Not assessed.
(71)	Cross sectional study	39 users, 28 non-users.	21.5 (2.3), 18-25	21.4 (2.0), 18-25	•None reported.	•Not assessed.
(69)	Cross sectional study	26 users, 10 non-users	Early-onset: 20.9 (2.9), late-onset: 22.2 (2.3)	SCZ: 20.4 (2.3), non-SCZ: 21.1 (2.8)	•Greater FA and white matter density in the left posterior corpus callosum in early-onset users compared to SCZ.	•Not assessed.
(70)	Cross sectional study	10 users, 10 non-users.	21.1 (2.9), 18-27	23.0 (4.4), 17-30	•Greater FA in the left anterior cingulate, left precentral gyrus, right medial frontal gyrus, left superior frontal gyrus, and right cingulate gyrus. •Greater apparent diffusion coefficient in left middle frontal gyrus and right posterior cingulate.	•Not assessed.
(74)	Cross sectional study	15 users, 15 non-users	25 (8.7)	25.2 (8.4)	•Decreased FA in left frontal lobe. •Greater overall diffusivity in right genu.	•Not assessed.
(66)	Cross sectional study	25 users, 18 non-users	23.16 (5.87)	23.11 (3.51)	•Decreased FA in the left internal capsule and bilateral genu of the corpus callosum.	•Not assessed.
(53)	Cross sectional study	19 users, 22 non-users.	20.58 (2.52), 18-25	21.59 (1.94), 18-25	•Decreased length of uncinate fasciculus fiber bundles.	•Not assessed.
(67)	Longitudinal study	158	BL: 20, FU: 22	BL: 20, FU: 20	•Attenuated developmental FA increase in the cingulum.	•Not assessed.
(54)	Cross sectional study	32 users, 43 non-users	21.6 (2.2), 18-26	21 (2.7), 16-25	•Decreased right uncinate fasciculus mean diffusivity.	•Greater cannabis use was associated with decreased FA in the forceps minor of males, while it was associated with increased FA in females.
(68)	Cross sectional study	33 users, 34 non-users	21.21, 18-25	21.12, 18-25	•Greater mean diffusivity in the uncinate fasciculi and corpus callosum forceps minor. •Decreased FA in uncinate fasciculi.	•Not assessed.

BL, baseline; FU, follow-up; FA, fractional anisotropy; RD, radial diffusivity; SCZ, patients with schizophrenia. *Changes included here were reported as statistically significant by the study’s author(s) (p <.05) unless otherwise stated.

**Table 4:** Summary of EEG studies.

Study	Study Design	Sample Size	Age of Users mean (SD), range	Age of non-users mean (SD), range	Changes associated with cannabis use*	Sex/Gender Differences*
(128)	Cross sectional study	21 users, 20 non-users	26.4, 18.5-52	24.7, 18.1-52.6	•Reduced P50 difference scores. •Greater P50 ratios.	•Not assessed.
(130)	Cross sectional study	18 users, 36 non-users	39.1, 20.8-56	MMN: 40.4, 21-52.6, P50: 31.2, 20.1-52.6	•None reported.	•Not assessed.
(129)	Cross sectional study	39 users, 42 non-users	26.4, 18.3-54.2	27.2, 18.1-52.6	•Decreased frequency (and duration in long-term users) mismatch negativity.	•Not assessed.
(131)	Cross sectional study	37 users, 38 smokers, 39 non-users.	21.7 (2.10), 18-25	Smokers: 21.4 (2.5), non-users: 22.1 (2.1), 18-25	•Decreased P3 ERP at the Cz, Fz, and FCz electrodes during a go/no-go task.	•Not assessed.
(132)	Cross sectional study	20 users, 84 non-users	F: 20.1 (1.2), M: 20.5 (1.2), 18-21.92	Controls: F: 19.9 (1.2), M: 20.0 (1.1) Alcohol users: F: 20.0 (1.2), M: 19.7 (1.2), 18-21.92	•Decreased amplitude of the N340 wavelength at the C1, C2, Cz, FC1, FCz, and FC2 electrodes during the Rey Auditory Verbal Learning Task.	•None found.

*Changes included here were reported as statistically significant by the study’s author(s) (p <.05) unless otherwise stated.

**Table 5:** Summary of MRS studies.

Study	Study Design	Sample Size	Age of Users mean (SD), range	Age of non-users mean (SD), range	Changes associated with cannabis use*	Sex/Gender Differences*
(75)	Cross sectional study	13 users, 25 users w/bipolar disorder (BP), 15 non-users, 14 non-users with bipolar disorder.	19.2 (1.1), BP: 18.4 (2.1)	18.4 (2.0), BP: 17.2 (3.0)	•Increased ventral lateral prefrontal cortex N-acetyl aspartate concentrations.	•Not assessed.
(79)	Cross sectional study	22 users, 21 non-users	25.05 (3.50), 18-34	24.24 (4.11), 19-33	•Decreased myoinositol in the hippocampus.	•None found.
(77)	Cross sectional study	27 users, 26 non-users	19.5 (0.6), 18-21	19.3(3.1), 13-24	•Increased myoinositol in the dorsal striatum. •Decreased dorsal striatum glutamate and glutamine.	•These effects were only observed in female participants.
(80)	Cross sectional study	18 users, 21 non-users	20.39 (0.99), 18-21	18.43 (2.57), 14-21	•Decreased anterior cingulate cortex GABA.	•Not assessed.

*Changes included here were reported as statistically significant by the study’s author(s) (p <.05) unless otherwise stated.

**Table 6:** Summary of studies utilizing other imaging methods.

Study	Study Design	Imaging modality	Sample Size	Age of Users mean (SD), range	Age of non-users mean (SD), range	Changes associated with cannabis use*	Sex/Gender Differences*
(81)	Cross sectional study	Computed transaxial tomography	12 users, 34 non-users	24.1, 20-30	25.8, 20-30	•None reported.	•Not assessed.

*Changes included here were reported as statistically significant by the study’s author(s) (p <.05) unless otherwise stated.

**Table 7:** Summary of PET studies.

Study	Study Design	Sample Size	Age of Users mean (SD), range	Age of non-users mean (SD), range	Changes significantly associated with cannabis use*	Sex/Gender Differences*
(133)	Cross sectional study	41 users, 18 non-users.	18.5 (0.6), 18-20	18.5 (0.6), 18-20	•Decreased [11C]ABP688 binding potential in the insula, ventral striatum, and medial orbitofrontal cortex	•Not assessed.
(134)	Cross sectional study	6 users, 6 non-users	20 (1), 18-21	20 (1), 18-21	•Decreased glucose metabolism in right orbitofrontal cortex, putamen, right cerebellum, and medial posterior parietal cortex.	•Not assessed.

*Changes included here were reported as statistically significant by the study’s author(s) (p <.05) unless otherwise stated.

**Table 8:** Summary of resting-state fMRI studies.

Study	Study Design	Sample Size	Age of Users mean (SD), range	Age of non-users mean (SD), range	Changes significantly associated with cannabis use*	Sex/Gender Differences*
(123)	Cross sectional study	17 users, 18 non-users	16.5 (0.2), 15-18	16.1 (0.4), 14-19	•Greater connectivity between the left cerebellum and bilateral inferior parietal lobules.	•Not assessed.
(124)	Longitudinal study	23 users, 21 non-users	BL: 19.4 (0.65), FU: 21.8 (0.81) 18-21	BL: 19.3 (1.18), FU: 21.5 (1.11) 18-21	•Absence of longitudinal growth in resting-state functional connectivity between the caudal anterior cingulate cortex and the right inferior parietal lobule and right precentral gyrus. •Diminished resting-state functional connectivity between the caudal anterior cingulate cortex and the right superior frontal gyrus and dorsal/rostral anterior cingulate cortex, and the bilateral medial dorsal and anterior thalamic nucleus.	•Not assessed.
(20)	Cross sectional study	70 users, 70 non-users	16–17 years or 26–29 years	16–17 years or 26–29 years	•Heightened resting-state connectivity in the executive control network.	•Not assessed.
(127)	Longitudinal study	23 users, 23 non-users	17.7 (0.7), 15-18	17.5 (0.8), 15-18	•Diminished cerebral blood flow in the left insula, bilateral medial frontal gyrus, left superior temporal gyrus, middle temporal gyrus, and the left supra-marginal gyrus. •Elevated cerebral blood flow in the right precuneus.	•Not assessed.
(119)	Cross sectional study	38 users, 37 non-users.	24.5 (1.3), ADHD: 24.7 (1.2), 21-25	24.4, ADHD: 25.3, 21-25	•Increased intrinsic functional connectivity in the left fusiform gyrus and right superior temporal sulcus.	•Not assessed.
(121)	Cross sectional study	43 users, 31 non-users	18 (1.22), 14-20	17.2 (1.38), 14-20	•Increased functional connectivity between the cingulate, right middle frontal gyrus, and the orbitofrontal cortex. •Greater functional connectivity between the orbitofrontal cortex and the left precentral gyrus and right superior frontal gyrus.	•Not assessed.
(120)	Cross sectional study	21 users, 21 non-users	23 (1.4), 20.8-25.6	22.9 (1.9), 20.5-26.8	•Increased effective connectivity between the left and right amygdala, left amygdala and anterior cingulate cortex, and right insula and medial prefrontal cortex.	•Not assessed.
(122)	Cross sectional study	17 users, 18 non-users	16.5 (0.2), 15-18	16.1 (0.4), 14-19	•Greater fractional amplitude of the low-frequency fluctuations (fALFF) activity in the right inferior frontal gyrus, right superior parietal gyrus, right inferior semilunar lobe of the cerebellum, right inferior temporal gyrus, and the right superior frontal gyrus. •Decreased interhemispheric connectivity in the superior frontal gyrus and cerebellar pyramis. •Greater interhemispheric connectivity in the supramarginal gyrus.	•Not assessed.
(126)	Cross sectional study	74	19.8 (1.62), 16-23	19.8 (1.62), 16-23	•Decreased functional connectivity between the default mode network and the prefrontal cortex. •Increased functional connectivity between the fusiform gyrus and the culmen and the default mode network. •Increased functional connectivity between the parahippocampal and temporal gyrus, caudate, and the default mode network.	•Not assessed.
(125)	Cross sectional study	8 users, 23 non-users	20.2 (2.19), 18-25	19.7 (1.42), 18-25, alcohol users: 19.54 (0.97), 18-25	•Reduced functional connectivity between the occipital fusiform gyrus and the left orbitofrontal cortex. •Greater functional connectivity between the right cerebellum and hippocampus.	•Not assessed.

*Changes included here were statistically significant (p <.05) unless otherwise stated.

**Table 9:** Summary of sMRI studies.

Study	Study Design	Sample Size	Age of Users mean (SD), range	Age of non-users mean (SD), range	Changes significantly associated with cannabis use*	Sex/Gender Differences*
(36)	Longitudinal study	799	BL: 14.4 (0.4), FU: 19.0 (0.7)	BL: 14.4 (0.4), FU: 19.0 (0.7)	•Decreased prefrontal cortex cortical thickness. •Increased rate of prefrontal cortex cortical thinning.	•None found.
(19)	Longitudinal study	2223	BL: 14-19, FU: 19-22	BL: 14-19, FU: 19-22	•Decreased cortical thickness in dorsomedial prefrontal cortex. •Increased rate of prefrontal cortex cortical thinning. •Change in cortical thickness in left lateral temporal cortex and heteromodal and medial paralimbic cortices.	•None found.
(45)	Cross sectional study	14 users, 14 non-users.	19.3 (0.8), 18-20	18.5 (1.4), 18-20	•Decreased size of hippocampus.	•Not assessed.
(46)	Cross sectional study	30 users, 29 non-users	21 (2.3), 18-30	22.4 (3.3), 18-30	•Gene-dependent changes in whole hippocampus and hippocampal subregion sizes.	•Not assessed.
(37)	Cross sectional study	24 users, 26 non-users	65.4 (7.2), 57-75	67.7 (7.1), 57-75	•Reduced cortical thickness of the whole hippocampus and and subregions.	•Not assessed.
(47)	Cross sectional study	18 users, 18 non-users	17.7 (0.94), 16-19	17.2 (0.82), 16-19	•Lower volume of the right medial orbital prefrontal cortex.	•Not assessed.
(48)	Cross sectional study	33 users, 42 non-users	21.3 (2.4), 18-25	21.9 (2.4), 18-25	•Increased volume of the cerebellar grey matter.	•None found.
(49)	Cross sectional study	11 users, 13 non-users	22 (2), 19-25	19-25	•Decreased grey matter volume in the right anterior hippocampus.	•Not assessed.
(50)	Longitudinal study	17 users, 17 non-users	20.7 (2.2), 18-25	21.5 (2.67), 18-25	•Greater longitudinal enlargement of- and total volumes of hippocampal subregions.	•None found.
(51)	Cross sectional study	20 users, 20 non-users	21.3 (1.9), 18-25	20.7 (1.9), 18-25	•Increased grey matter density in the hypothalamus, left nucleus accumbens to subcallosal cortex, left amygdala, and sublenticular extended amygdala. •Absence of a correlation between nucleus accumbens grey matter density and morphological features. •Altered shape of left nucleus accumbens and right amygdala.	•Not assessed.
(52)	Cross sectional study	20 users, 20 non-users	21.4 (2.0), 18-25	20.4 (1.7), 18-25	•Increased grey matter density in the left nucleus accumbens.	•Not assessed.
(62)	Longitudinal study	20 users, 23 non-users	BL: 20.64 (2.23), 18-24, FU: 25.07 (2.64)	BL: 21.79 (2.6), 18-24, FU: 25.07 (2.64)	•None reported.	•Not assessed.
(53)	Cross sectional study	19 users, 22 non-users.	20.58 (2.52), 18-25	21.59 (1.94), 18-25	•Decreased cortical thickness in the right amygdala, fusiform gyrus, and entorhinal cortex.	•Not assessed.
(38)	Cross sectional study	55 users, 65 non-users	23.6 (1.5), ADHD: 24.3 (1.3)	23.4 (1.5), ADHD: 24.6 (1.4)	•Cortical thinning in right anterior/posterior cingulate cortex, left precentral gyrus, and bilateral superior frontal gyrus.	•Not assessed.
(39)	Cross sectional study	18 users, 18 non-users	17.8 (1.0), 16-19	17.3 (0.8), 16-19	•Cortical thinning in the bilateral insula, bilateral superior frontal cortex, and the right caudal middle frontal cortex. •Cortical thickening in the right inferior and superior parietal cortex, bilateral lingual gyrus, paracentral sulcus, and the right superior temporal cortex.	•Not assessed.
(54)	Cross sectional study	32 users, 43 non-users	21.6 (2.2), 18-26	21 (2.7), 16-25	•Increased volume of the right superior temporal lobe.	•Decreased volume of the left rostral anterior cingulate cortex was only observed in females.
(40)	Cross sectional study	30 users, 44 non-users	25.7 (5)	25.8 (5.8)	•Reduced gyrification in left temporal and bilateral frontal lobes. •Absence of longitudinal reductions in frontal lobe cortical thickness and gyrification.	•Not assessed.
(55)	Cross sectional study	35 users, 47 non-users	Female: 18.15 (0.86), 16-18, male: 17.92 (0.91), 16-18	Female: 17.85 (0.73), 16-18, male: 17.65 (0.90), 16-18	•Volume increase in the amygdala.	•This effect was only reported in females. No difference in males.
(61)	Cross sectional study	16 users, 16 non-users	18.7 (0.7), 16-18	18.9 (0.9), 16-18	•None reported.	•Not assessed.
(63)	Cross sectional study	16 users, 16 non-users	Females: 18.2 (0.6), 16-18, males: 18.1 (0.8), 16-18	Females: 18.5 (0.5), 16-18, males: 17.7 (1.1), 16-18	•None reported.	•Trend-level interaction between gender and cannabis use, such that male users had decreased prefrontal cortex volumes, while the opposite was observed in females.
(56)	Cross sectional study	16 users, 16 non-users	18.11 (0.74), 16-18	18.01 (0.97), 16-18	•Higher volume of the inferior posterior vermis.	•None found.
(44)	Longitudinal study	94 users, 87 non-users	Youngest cohort: 29.6 (1.23), oldest cohort: 36.2 (1.52)	Youngest cohort: 29.6 (1.23), oldest cohort: 36.2 (1.52)	•None reported.	•Not assessed.
(41)	Longitudinal study	537	BL: 14, FU: 19	BL: 14, FU: 19	•Increased rate of longitudinal cortical thinning.	•Not assessed.
(57)	Cross sectional study	27 users, 32 non-users	21.41 (2.21), 18-25	21.09 (2.32), 18-25	•Decreased volume of the medial orbitofrontal cortex.	•None found.
(42)	Cross sectional study	41 users, 41 non-users	20.3 (2.1), 18-25, Major Depressive Disorder: 21.3 (2.4), 18-25	21.5 (2.0), 18-25, Major Depressive Disorder: 22.1 (2.5), 18-25	•Cortical thinning in the right entorhinal cortex, middle temporal gyrus, medial orbitofrontal cortex, and superior frontal gyrus.	•Not assessed.
(43)	Cross sectional study	147 users, 634 non-users	Occasional: 18.1 (2), 14-22, frequent: 18.5 (1.6), 15-22	17 (2.1), 14-22	•Slight cortical thinning in the left frontal lobe.**	•Not assessed.
(58)	Cross sectional study	33 users, 35 non-users	21.21 (2.34), 18-25	21.14 (2.33), 18-25	•Gyrification reductions in the frontal lobes and ventral-medial and medial prefrontal cortex.	•Not assessed.
(60)	Cross sectional study	79 users, 79 non-users	adolescents: 16.65 (1.09), 14-18, adults: 27.4 (7.1), 18-53	adolescents: 16.77 (0.95), 14-18, adults: 27.5 (6.8), 18-53	•None reported.	•Not assessed.
(59)	Longitudinal study	706	BL: 14.41 (0.93), FU: 18.89 (0.66)	BL: 14.41 (0.93), FU: 18.89 (0.66)	•Attenuated longitudinal increase in the volume of the uncus.	•None found.

*Changes included here were reported as statistically significant by the study’s author(s) (p <.05) unless otherwise stated. **Scott et al.’s (43) reported finding was significant but not reported as being reflective of cannabis-related damage.

**Table 10:** Summary of task-based fMRI studies.

Study	Study Design	Sample Size	Age of Users mean (SD), range	Age of Non-Users mean (SD), range	Task Paradigm	Changes significantly associated with cannabis use*	Sex/Gender Differences*
(104)	Cross sectional study	14 users, 14 non-users	17.6 (1), 15-19	17.3 (1.3), 15-19	Digital coin flip	•Increased activity in the claustrum, middle frontal gyri, and caudate when winning. •Increased activity in the claustrum, declive, right posterior and anterior cingulate, right middle frontal gyrus, and the left insula when losing.	•Not assessed.
(82)	Cross sectional study	82	16.2 (1.2), 14-18	15.6 (1.37), subclinical use: 16.6 (1.34), AUDIT ≥ 4: 16.5 (1.17) 14-18	Affective Stroop task	•Greater activation in the precuneus, inferior parietal lobule, and the posterior cingulate cortex during incongruent trials.	•Not assessed.
(86)	Cross sectional study	13 users, 15 non-users	21.15 (1.9), 19-25	20.27 (2.3), 18-25	Implicit association test	•Increased activity in the putamen, caudate, and right inferior frontal gyrus during compatible trials. •Decreased activity in the prefrontal cortex and right inferior frontal gyrus during incompatible trials.	•Not assessed.
(100)	Cross sectional study	21 users, 21 non-users	24.95 (3.56), 18-34	24.24 (4.11), 19-33	Paired associates task	•Absence of activity change in the left posterior cingulate gyrus and right precuneus during repeated recall. •Alterations in activity trajectories across areas of the midbrain over blocks of recall.	•Not assessed.
(92)	Cross sectional study	39 users, 150 non-users	15.97 (1.06), 14-18	16.05 (1.18), alcohol users: 16.35 (1.11), alcohol and cannabis users: 16.31 (1.09) 14-18	Balloon analogue risk task	•None reported.	•Not assessed.
(106)	Longitudinal study	32 users, 41 non-users	BL: 21.4 (2.3), FU: 21.9 (2.4)	BL: 22.2 (2.4), FU: 22.7 (2.4)	Iowa gambling task	•Increased activity in the right insula, left superior temporal gyrus, and orbitofrontal cortex during wins.	•Not assessed.
(117)	Cross sectional study	53 users, 22 non-users	Frequent users: 21.3 (2.3), sporadic users: 22.1 (2.5), 18-25	22.1 (2.4), 18-25	Cue-reactivity task	•Increased activity in the ventral tegmental area, anterior cingulate cortex, dorsal/ventral striatum, orbitofrontal cortex, and medial frontal cortex when viewing cannabis-related images.	•Not assessed.
(103)	Longitudinal study	26 users, 23 non-users	BL: 21 (2.3), 18-25, abstinent users: 22.4 (3.6), FU: 24.5 (2.6), abstinent users: 24.7 (2.9)	BL: 22.1 (2.5), 18-25, FU: 25.3 (2.5)	N-back task	•None reported.	•Not assessed.
(102)	Longitudinal study	32 users, 41 non-users	BL: 21.4 (2.4), FU: 21.6 (2.4)	BL: 22 (2.3), FU: 22.5 (2.4)	N-back task	•None reported.	•Not assessed.
(89)	Cross sectional study	28 users, 32 non-users	19.3 (2), 14-23	18.9 (2.7), 14-23	Flanker task	•Absence of increased activation in the ventromedial prefrontal cortex, precentral gyrus, dorsal/ventral prefrontal cortex, parietal lobe, pallidum, left paracentral lobule, occipital gyri, right putamen, thalamus, middle cingulate, and precuneus during incongruent trials.	•Not assessed.
(98)	Cross sectional study	27 users, 33 non-users	18.3 (0.5), 18-22	18.4 (0.6), 18-22	Figural memory task	•Absence of activation of the hippocampus during encoding. •Absence of activation in the left inferior frontal gyrus during encoding. •Decreased activation of the parahippocampal gyri, left basal ganglia, left insula, cerebellum, and the right precentral gyrus during encoding. •Decreased activation of the hippocampus and left inferior frontal gyrus during correct responding.	•Activity in the left inferior frontal gyrus, left hippocampus, and the right inferior frontal gyrus was lower in males during responding, but unchanged in females.
(112)	Cross sectional study	20 users, 22 non-users	20.6 (2.5), 18-25	21.5 (1.9), 18-25	Cyberball	•Absence of exclusion-evoked activity in the insula.	•Not assessed.
(52)	Cross sectional study	20 users, 20 non-users	21.4 (2.0), 18-25	20.4 (1.7), 18-25	Social influence task	•More diffuse activation of the striatum during the social influence condition. •Increased activity of the nucleus accumbens when accepting the group’s choice.	•Not assessed.
(113)	Cross sectional study	20 users, 23 non-users	20.6 (2.5), 18-25	21.6 (1.9), 18-25	Social influence task	•Increased activity in the caudate during the social influence condition. •Greater inferior frontal gyrus activation when rejecting the group’s choice.	•Not assessed.
(90)	Cross sectional study	23 users, 16 non-users	22.43 (5.29)	22.75 (2.82)	Multi-source interference task	•Smaller region of activation within the anterior cingulate cortex in response to incongruent stimuli.	•Not assessed.
(85)	Cross sectional study	21 users, 21 non-users	36.5 (8.8)	31 (11.7)	Multi-source interference task	•Greater functional connectivity between the anterior insular cortex, occipitoparietal cortex, dorsal anterior cingulate, and the lateral prefrontal cortex with increasing task difficulty.	•Not assessed.
(21)	Cross sectional study	10 users, 14 non-users	19-21	19-21	Counting Stroop task	•Greater activity in the right postcentral gyrus, right rolandic operculum, and the right cerebellar tonsil, right supplementary motor area, cingulate gyrus, and left postcentral gyrus during incongruent trials.	•Not assessed.
(114)	Longitudinal study	20 users, 20 non-users	19.84 (1.45), 17-22	20.51 (1.26), 17-22	Emotion-arousal word task	•Decreased activity in the right middle and superior temporal gyri, amygdala, right calcarine fissure + lingual gyri and cuneus, right superior temporal gyrus and insula, and the right middle frontal and dorsolateral superior frontal gyri during the presentation of negative words. •Increased activity in the right dorsolateral superior frontal gyrus and decreased activity in the right inferior parietal lobe and amygdala during the presentation of positive words.	•Not assessed.
(101)	Cross sectional study	135	16.5 (0.13), 14-18	16.5 (0.14), Alcohol users: 16.9 (0.16), 14-18	Passive avoidance task	•Delayed increase in functional connectivity between frontoparietal and striatal regions as learning took place.	•Not assessed.
(110)	Cross sectional study	132	15.79 (1.4), 14-18	15.76 (1.2), tobacco users: 16.29 (1.2), cannabis and tobacco users: 15.76 (1.15), alcohol users: 16.00 (1.23), cannabis, tobacco, and alcohol users: 15.94 (0.97), 14-18	Monetary incentive delay task	•None reported.	•Not assessed.
(91)	Cross sectional study	36 users, 33 non-users	Median: 21, 18-25	Median: 21, 18-25	N-back flanker task	•Absence of greater superior temporal gyrus activity during progressively harder trials. •Reduced activity in the postcentral gyrus, superior parietal lobe, insula, operculum, supramarginal gyrus, and thalamus during trials with cannabis-related flankers.	•Not assessed.
(109)	Longitudinal study	47 users, 111 non-users	Stable-high use: 20.09 (0.35), escalating use: 20.07 (0.24)	20.10 (0.26)	“card-guessing game”	•Inversely correlated activation patterns in the nucleus accumbens and medial prefrontal cortex during reward processing in those with increasing use during adolescence. Opposite of what was seen in consistently high users and non/low users.	•Not assessed.
(118)	Cross sectional study	24 users, 24 non-users	18.2 (0.7), 16-19	18 (1.9), 16-19	Finger-tapping task	•Decreased activity in the cerebellum and right cingulate gyrus.	•Not assessed.
(111)	Longitudinal study	164 users, 154 non-users	19-22	19-22	Monetary incentive delay task	•None reported.	•Not assessed.
(87)	Cross sectional study	34 users, 32 non-users	female: 21.4 (20), 19-25, male: 21.7 (2.0), 18-25	female: 21.2 (2.4), 18-25, male: 20.9 (2.7), 16-25	Go/no-go task w/emotional stimuli	•Decreased activity in the rostral anterior cingulate cortex when inhibiting responses to fearful faces.	•None found.
(108)	Longitudinal study	108	BL: 20.1 (1.4), FU1: 22.1 (1.5), FU2: 23.8 (1.7)	BL: 20.1 (1.4), FU1: 22.1 (1.5), FU2: 23.8 (1.7)	Modified monetary incentive delay task	•Reduced nucleus accumbens activity in response to the expectation of reward.	•Not assessed.
(107)	Cross sectional study	18 users, 18 non-users	16.11 (0.41)	16.5 (0.23)	Monetary incentive delay task	•Increased connectivity between the orbitofrontal cortex, amygdala, nucleus accumbens, lateral/medial prefrontal cortex, hippocampus, and temporal cortex while expecting reward.	•Not assessed.
(115)	Cross sectional study	37 users, 40 non-users	19.85 (1.63), 16-23	19.85 (1.63), 16-23	Emotion regulation task	•Greater left superior temporal gyrus activity in response to positive trials and reduced activity in response to negative trials.	•Not assessed.
(97)	Cross sectional study	17 users, 17 non-users	18.06 (0.75)	17.9 (1.12)	Spatial working memory task	•Increased activity in the parietal lobes and the right basal ganglia during the spatial working memory trials. •Increasing activity in the left superior temporal gyrus and decreasing activity in the right superior temporal gyrus with more accurate responses. •Increased activity in the left anterior cingulate cortex with more accurate responses. •Decreased activity in the pulvinar and right thalamus with more accurate responses.	•Not assessed.
(83)	Cross sectional study	50 users, 34 non-users	23.98 (6.95), 17-46	24.53 (6.57), 17-46	Stroop color word test	•Increased activity in the left anterior cingulate cortex during incompatible trials.	•Not assessed.
(94)	Cross sectional study	15 users, 17 non-users	18.1 (0.7), 16-18	17.9 (1.0), 16-18	Spatial working memory task	•Increased activity in the inferior cuneus and right superior parietal lobule during spatial working memory trials. •Reduced activity in the medial right superior cuneus and right dorsolateral prefrontal cortex during spatial working memory trials	•Not assessed.
(99)	Cross sectional study	8 users, 64 non-users	18.1 (0.9), 16-18	17.6 (0.8), binge drinkers: 18.1 (0.7), binge drinker and users: 18.0 (1.0), 16-18	Paired associates task	•Absence of hippocampus activity while encoding. •Increased activity of regions in the frontal lobe while encoding. •Decreased activity in the lingual gyrus and cuneus while encoding.	•None found.
(105)	Cross sectional study	63 users, 62 non-users	Adolescents: 17.22 (0.52), 16.31-17.98, adults: 27.81 (1.49), 26.27-30.02	Adolescents: 17.15 (0.45), 16.27-18.04, adults: 27.34 (0.86), 26.10-29.56	Monetary incentive delay task	•Increased activity in the right frontal pole and inferior parietal cortex during reward.	•Not assessed.
(95)	Cross sectional study	10 users, 14 non-users	20, 19-21	20, 19-21	Visuospatial 2-back task	•Increased activity in the right superior temporal gyrus, right inferior frontal gyrus, and the left middle frontal gyrus during 2-back trials.	•Not assessed.
(93)	Cross sectional study	10 users, 14 non-users	20, 19-21	20, 19-21	Go/no-go task	•None reported.	•Not assessed.
(116)	Longitudinal study	578	BL: 14.6 (0.42) FU: 19.4 (0.96)	BL: 14.4 (0.42) matched controls: 14.5 (0.37), FU: 18.9 (0.67) matched controls: 19.3 (0.65)	Face processing task	•Increased activity in the right amygdala on trials with angry faces. •Absence of longitudinal increases in right amygdala activity in response to angry faces.	•Not assessed.
(88)	Cross sectional study	34 users, 32 non-users	Female: 21.4 (2.0), 19–25 male: 21.7 (2.0), 18-25	Female: 21.2 (2.4), 18–25 male: 20.9 (2.7), 16-25	Go/no-go task w/emotional faces	•Reduced rostral anterior cingulate cortex activity when having to inhibit responses to fearful faces.	•Increased connectivity between the cerebellum and the right rostral anterior cingulate cortex in males.
(84)	Cross sectional study	16 users, 17 non-users	18.1 (0.7), 16-18.9	17.9 (1), 16-28.9	Go/no-go task	•Increased activity in the right superior middle frontal gyrus, posterior parietal cortex, right lingual gyrus, right middle frontal gyrus, anterior insula, left anterior middle gyrus, right anterior superior gyrus, superior frontal gyrus, and medial prefrontal cortex during no-go trials. •Increased activity in the medial precuneus, right inferior frontal gyrus, right superior parietal lobule, anterior insula, right superior frontal gyrus, right inferior parietal lobule during go-trials.	•Not assessed.
(96)	Prospective cohort study	22 users, 63 non-users	15.58 (0.35)	15.65 (0.37)	Spatial working memory task	•Reduced activity in the cuneus.	•Not assessed.

*Changes included here were statistically significant (p <.05) unless otherwise stated.

### 3.2 Structural neuroimaging results

#### 3.2.1 sMRI

##### 3.2.1.1 Cortical thickness

We categorized sMRI findings based on the morphological measure examined, here being cortical thickness or volume. Of the included sMRI papers, 38% (10/26) examined cortical thickness with 80% (8/10) reporting decreased cortical thickness in cannabis using AYA compared to non-using controls (19, 36–42). Cortical thinning was usually located within the frontal lobes (19, 36, 40, 42), the hippocampus (37) and the cingulate (38). Two studies found no evidence of cortical thinning related to cannabis use (43, 44).

##### 3.2.1.2 Volume changes

Of the sMRI papers assessing brain volumes, 71% (15/21) found significant regional volume differences between users and non-users (45–59). The most common area of change reported was in the hippocampus, however, there was no consensus on change direction as both increases and decreases in volume were reported (45, 46, 49, 50). AYA cannabis use was only associated with decreasing volumes in the prefrontal cortex (47, 57, 58) with the amygdala showing evidence of volume increase in two studies (51, 55) but a decrease in another (53). Other brain areas reporting AYA cannabis related volume changes include the cerebellum [volume increase; (48, 56)], the nucleus accumbens [volume increase; (52)], and the uncus [absence of longitudinal volume growth; (59)]. The remaining 29% of the included studies found no significant effect of AYA cannabis use on brain volumes (43, 44, 60–63).

##### 3.2.1.3 Abstinence in sMRI

Medina et al. (2010) reported that cerebellar volume increases related to cannabis use remained after a month of abstinence, Ashtari et al. (2011) reported on cannabis-related changes in hippocampal volumes which persisted after an average of 6.7 months of abstinence, and Burggren et al. (2018) found changes in hippocampal cortical thickness persisted after a minimum of 22 years of abstinence from regular use (37, 45, 56). However, Medina et al. (2009) found no abnormalities in prefrontal cortex volumes in cannabis users who had been abstinent for 1 month (63).

##### 3.2.1.4 Sex/gender differences in sMRI

Possible sex/gender effects have been reported by McQueeny et al. (2011), who found cannabis associated increases in amygdala volume only in women (55). Maple (2016) had a similar finding, observing that only women cannabis users showed a significant decrease in the volume of the left rostral anterior cingulate cortex (54). Medina et al. (2009) reported that cannabis use tended to be associated with increased prefrontal cortex volume in women with the opposite being found in men, however this gender effect was not statistically significant (63).

##### 3.2.1.5 Summary of sMRI findings

Taken together, the weight of the current evidence seems to suggest that AYA cannabis use is associated with cortical thinning, particularly in the frontal lobes. Volume changes related to AYA cannabis use were inconsistent in their direction, being commonly associated with both volume increases and decreases in the hippocampus and volume decreases in the prefrontal cortex. Additionally, the weight of this small body of literature seems to suggest that structural brain changes related to cannabis use in young adults are potentially persistent. However, more research and longer periods of abstinence are needed to bolster the strength of this claim. Due to the relatively few studies which explicitly assessed sex/gender effects, research regarding sex/gender differences in the impact of AYA cannabis use on sMRI measures should be pursued.

#### 3.2.2 DTI

##### 3.2.2.1 Fractional anisotropy changes

Of the DTI papers included in this review, 9/12 assessed fractional anisotropy (FA) as the primary DTI variable, a broad measure of the integrity of neuronal pathways. Approximately 56% (5/9) of these papers found lower FA values across the brain (64–68), indicating a loss of neuronal integrity from possible neuron/myelin abnormalities, or in axon packing/coherence. Other researchers have reported increased FA in multiple regions of the brain (69, 70)), hypothesized to indicate potential hyperconnectivity between brain regions. However, two studies have found no differences in FA between AYA users and non-users (23, 71). Furthermore, FA has been criticized to be too broad of a measure, limiting the interpretation of these findings (72).

##### 3.2.2.2 Other DTI metrics

DTI can generate other indices of neuronal integrity. However, within the included DTI studies, there was a great deal of heterogeneity regarding the measures used and the areas assessed. Ashtari et al. (2009) found decreased axial diffusivity in the motor pathways and increased radial diffusivity and apparent diffusion coefficient in the arcuate fasciculus (64). Becker et al. (2015) found changes in radial diffusion in multiple subcortical areas, reflecting potential cannabis-related changes to neuronal microstructure (65, 73). Gruber et al. (2011) found greater overall diffusivity in the right genu, which also potentially reflects neuronal degradation (73, 74). Both Maple (2016) and Shollenbarger et al. (2015b) reported that the uncinate fasciculus had decreased mean diffusivity in AYA cannabis users (54, 68) and Levar et al. (2018) found that fiber bundles in the uncincate fasciculus were significantly shorter in AYA cannabis users than in non-using controls (53). Interestingly, DeLisi et al. (2006) found a greater apparent diffusion coefficient in the frontal and subcortical areas of cannabis users compared to controls, which contrasts others findings (70).

##### 3.2.2.3 Sex/gender differences in DTI

Of the included DTI studies, only Maple (2016) examined sex/gender differences, reporting decreased FA in the forceps minor of AYA cannabis using men, while finding increased FA in AYA cannabis using women (54).

##### 3.2.2.4 Abstinence

Ashtari et al.’s (2009) finding of cannabis use-related changes in FA were documented in users who were abstinent for at least 3 months (*M* = 6.7 months) (64). Shollenbarger et al. (2015b) also found that the cannabis-related change in mean diffusivity of the uncinate fasciculus persisted after a week of abstinence (68).

##### 3.2.2.5 Summary of DTI findings

Despite the wide-ranging results included in this section, collectively they indicate that cannabis use during AYA is associated with changes to the microstructure of the brain. Many of the included studies found that AYA cannabis use was associated with changes in the FA of multiple brain areas, loosely suggesting cannabis-related neuronal hyper- and hypoconnectivity. Other, more robust measures of neuronal microstructure were also used by the included studies to support the notion that AYA cannabis use is associated with changes to neuronal microstructure. Regarding sex/gender differences and abstinence effects in the DTI literature, very little can be concluded overall due to the paucity of relevant reporting on the topic, highlighting the need for future researchers to consider these constructs in their studies of AYA cannabis use and neuronal microstructure.

#### 3.2.3 MRS

Of the relatively few (*n* = 4) MRS studies included, there were no significant themes or overlapping results. Bitter et al. (2014) found that cannabis users had an increased concentration of N-acetyl aspartate (NAA) in the ventral lateral prefrontal cortex, a marker of neuronal integrity and potentially increased neuronal density (75, 76). Muetzel et al. (2013) found increased concentrations of myoinositol in the dorsal striatum in cannabis users, potentially indicating neuroinflammation (76, 77). In the same study, Muetzel et al. (2013) also found lower concentrations of glutamine and glutamate in the dorsal striatum, potentially indicating decreased energy metabolism (78) and deficits in excitatory neurotransmission (76) in AYA cannabis users compared to non-users (77). Interestingly, Blest-Hopley et al. (2020) found decreased myoinositol in the hippocampus, again potentially indicating a neuroinflammation process in this region (76, 79). Finally, Subramaniam et al. (2022) found decreased GABA in the anterior cingulate cortex, indicating a deficit in inhibitory neurotransmission (78, 80).

##### 3.2.3.1 Sex/gender differences in MRS

Only 2 of the included MRS studies explicitly assessed sex and/or gender differences related to the impact of AYA cannabis use on the brain (77, 79). Of these, only Muetzel et al. (2013) reported a significant effect. Specifically, their finding of increased myoinositol and decreased glutamate and glutamine in the dorsal striatum was only present in female participants (77).

##### 3.2.3.2 Summary of MRS findings

Again, due to the few MRS studies included in this review and their lack of uniformity regarding brain areas and metabolites of interest, there is no way to meaningfully abstract across these results. The limited number of studies assessing sex/gender and the complete lack of included studies explicitly assessing abstinence also make these constructs an important area of future research.

#### 3.2.4 Other structural imaging modalities

The remaining structural imaging study utilized computed transaxial tomography (CT). In this early study, Co et al. (1977) reported no effect of cannabis use on any morphological brain measure in this cohort (81).

### 3.3 Functional neuroimaging

#### 3.3.1 Task-based fMRI

##### 3.3.1.1 Cognitive control

Of the included task-based fMRI studies, 31% (12/39) found that cannabis users’ brains responded significantly differently from non-users during tasks requiring cognitive control or decision making. During tasks requiring greater than normal levels of cognitive control (e.g., incongruent trials in the Stroop task or “no-go” trials in a go/no-go task), 10% of the included studies found that AYA cannabis use was associated with heightened activation in a number of brain regions compared to non-using controls (21, 82–84). Similarly, with increasing task difficulty, Harding et al. (2012) found greater functional connectivity between multiple brain areas (85). However, other studies found that trials requiring cognitive control elicited less activity throughout the brains of AYA cannabis users compared to non-users. (86–88). Cyr et al. (2019) found that multiple brain regions which showed elevated activation in controls during trials requiring cognitive control showed no changes in cannabis users and Gruber et al. (2012) found that a smaller area of the anterior cingulate cortex (ACC) was activated in cannabis users compared to controls on trials of this type (89, 90). Adding to this, Kroon et al. (2022) found that task difficulty increases were not associated with increased activation of the superior temporal gyrus in cannabis users (91)). Only one study found an effect of cannabis use on brain activity during trials which did not require above-average cognitive exertion (i.e., congruent trials in the Stroop task or “go” trials in a go/no-go task). In this study, Ames et al. (2013) found that cannabis users had greater activity in an array of brain areas during compatible trials of an implicit association task (86). However, both Claus et al. (2018) and Smith et al. (2011) found no differences in the activation patterns of the brains of cannabis users and non-users during tasks of cognitive control (92, 93).

##### 3.3.1.2 Learning and memory

The next most common fMRI task type was those which assessed learning and/or memory in some capacity (11/39). In a variety of memory tasks, the included studies found that AYA cannabis users displayed patterns of activation that significantly differed from the brains of non-users (94–97), with no particular pattern predominating. With respect to specific stages of memory, cannabis use was found to be associated with an absence of hippocampal activation during the encoding of new memories in two studies (98, 99). In addition, these studies found that cannabis use was associated significantly with altered patterns of activity across other areas of the brain during encoding, such as the frontal lobes (98, 99). During the recall of previously encoded information, Blest-Hopley et al. (2021) found no related activity change in the left posterior cingulate gyrus and right precuneus alongside alterations in the patterns of activation in midbrain structures (100). Further, Dager et al. (2018) found decreased recall-related activity in cannabis users (98). Regarding learning, Hubbard et al. (2023) observed a delayed increase in functional connectivity between frontoparietal and striatal regions as learning took place in cannabis users (101). In opposition, as part of a single longitudinal study, Cousijn et al. (2014a) and Cousijn et al. (2014b) reported no cannabis-related changes in brain function during memory tasks (102, 103). Considered together, these results suggest that there likely exists changes to memory functioning attributable to AYA cannabis use.

##### 3.3.1.3 Reward

The third most common type (8/39) of task-based fMRI studies assessed risk and reward systems. The first observable trend among these studies was that cannabis users displayed significantly increased activation in response to rewarding stimuli compared to non-users (104–106). Similarly, Nestor et al. (2020) found that cannabis users had increased functional connectivity across the brain when expecting reward (107). These studies suggest that the brains of AYA cannabis users are more sensitive to reward. Unlike these studies, Martz et al. (2016) observed that cannabis users had decreased activity in the nucleus accumbens when expecting reward (108). Lichenstein et al. (2017) found that cannabis users who increased their use throughout adolescence exhibited a pattern of brain activity opposite to non/low users and consistently high users when experiencing reward (109). Specifically, cannabis users who increased their use between the ages of 14–19 displayed inversely correlated patterns of activity between the nucleus accumbens and prefrontal cortex when winning in a card guessing game at age 20, as opposed to non/low users and consistently high users which displayed a direct relationship between nucleus accumbens and prefrontal cortex activation (109). Contrary to these studies, Karoly et al. (2015) and Macedo et al. (2024) found that AYA cannabis use was not associated with any changes in reward processing (110, 111).

##### 3.3.1.4 Social cognition

Only a small number (3/39) of the included studies assessed how cannabis use impacts social cognition. Gilman et al. (2016a) found that cannabis users lacked the usual elevated activation of the insula when being socially excluded (112). Further research has found the opposite, namely that tasks of social cognition elicit greater brain activity in cannabis users: Gilman et al. (2016b) found that a greater area of the striatum was activated by cannabis users when they were being socially influenced and that cannabis users had an increase in nucleus accumbens activity when succumbing to social influence relative to non-users (52). Finally, Gilman et al. (2016c) observed increased caudate activation in cannabis users during social influence generally and greater inferior frontal gyrus activity when rejecting social influence (113).

##### 3.3.1.5 Emotion processing

Another small portion of included fMRI studies (3/39) investigated how cannabis use impacts the processing of emotion. Heitzeg et al. (2015) found that cannabis users displayed decreased activity in many areas when viewing negatively valanced words while also displaying increased activity when viewing positively valanced words (114). Nichols et al. (2021) made a similar observation, finding that cannabis users had greater activity in the left superior temporal gyrus in response to positive stimuli and less activity in response to negative stimuli (115). Spechler et al. (2020) found that cannabis use was associated with increased activity in the right amygdala when presented with angry faces at a baseline assessment and that cannabis users displayed no longitudinal increase in this response compared to non-users (116).

##### 3.3.1.6 Other tasks

In a cue-reactivity task, Cousijn et al. (2013a) presented cannabis users and non-users with cannabis-related images while undergoing a fMRI, finding that cannabis users displayed greater brain activity in multiple regions when viewing cannabis-related images (117). Lopez-Larson et al. (2012) had cannabis users and non-users do a finger-tapping task and found that it elicited decreased activity in the cerebellum and right cingulate gyrus of cannabis users compared to non-users (118).

##### 3.3.1.7 Abstinence

Regarding the effects of abstinence on cannabis-related task based functional changes, Macedo et al. (2024) found no abnormalities in brain activity in <1-month abstinent users undergoing a monetary incentive delay task, but also found no effect in current users, so the effect of abstinence on changes in reward processing in this case is unclear (111). Within the included studies, abnormalities in brain activity during tasks of spatial working memory (94, 97) and response inhibition (84) were still observable after 28 days of abstinence. Finally, Sullivan et al. (2022) found blunted activation in the anterior cingulate cortex in response to fearful faces in cannabis users who were abstinent for 2 weeks (88).

##### 3.3.1.8 Sex/gender differences in task-based fMRI

Dager et al. (2018) reported a sex effect in that only AYA cannabis using males displayed reduced activity in the bilateral inferior frontal gyrus and left hippocampus when correctly responding to a figural memory task (98). Sullivan et al. (2022) found, compared to women, AYA cannabis users who were men had significantly greater connectivity between the cerebellum and right rostral anterior cingulate cortex during a go/no-go task with emotional faces, which made their brain activity more in-line with non-users (88).

##### 3.3.1.9 Summary of fMRI findings

Abstracting across the included fMRI studies makes it clear that AYA cannabis use is likely associated with a vast array of changes in brain activity during cognitive tasks. Significant increases and decreases in activity in a variety of brain areas were found during tasks of cognitive control, learning and memory, reward, social cognition, emotion processing, cue reactivity, and motor control. The fMRI studies with explicitly abstinent AYA cannabis users suggest that cannabis-associated changes in brain activity persist after 2 weeks to a month of non-use. Regarding sex/gender differences, due to the lack of studies explicitly assessing sex/gender and the heterogenous results of those which did, very little can be concluded until further evidence comes forward.

#### 3.3.2 Resting-state fMRI

60% (6/10) of the included rs-fMRI studies found increased connectivity between many regions of the brain at rest in cannabis users (20, 119–123). Other researchers have found that cannabis users exhibit decreased connectivity between a variety of regions of the brain (124–126). Common areas of change include the default mode network and (119, 126), and the orbitofrontal cortex (121, 125).

##### 3.3.2.1 Abstinence in rs-fMRI

Jacobus et al. (2012) found significant differences in cerebral blood flow at baseline in AYA cannabis users compared to non-users, but this difference disappeared after the users underwent 4 weeks of abstinence (127).

##### 3.3.2.2 Summary of rs-fMRI findings

Across the included studies, it appears clear that AYA cannabis use is associated with changes to the resting-state activity of the brain, particularly the default mode network and orbitofrontal cortex where both increases and decreases in activity have been observed. Following a trend present in other imaging modalities, very little re-fMRI evidence regarding the effects of sex/gender or abstinence exists, meaning these constructs should be the focus of future research.

#### 3.3.3 EEG

Broyd et al. (2013) found that AYA cannabis users displayed diminished sensory gating and Greenwood et al. (2014) found significant abnormalities in EEG signals related to mismatch negativity (MMN) in AYA cannabis users (128, 129). Broyd et al. (2016) replicated their sensory gating study, although findings were only different at a trend level (*p*>.05) (130). Apart from these findings, Maij et al. (2017) found that AYA cannabis users exhibited a significantly lower average amplitude of the P3 ERP at the Cz, Fz, and FCz electrodes during a go/no-go task, which the authors interpret as indicating deficient response inhibition (131). Finally, regarding memory, Smith et al. (2017) reported that AYA cannabis users had significantly lower N340 amplitude at electrodes C1, C2, Cz, FC1, FCz, and FC2 during the Rey Auditory Verbal Learning Task, indicating deficient memory (132).

##### 3.3.3.1 Abstinence studies with EEG

The only EEG study to explicitly examine abstinent users was Broyd et al. (2016), reporting no significant abnormalities in ERPs relating to sensory processing in AYA cannabis users who had been abstinent for >1 month [*M* = 3.5 years; (130)]. Since Broyd et al. (2016) did not also make these measurements prior to abstinence nor compare these users to current cannabis users, the strength of this finding alone is questionable. However, when viewed in the context of Broyd et al.’s (2013) finding of deficiencies in sensory gating in *current* cannabis users, Broyd et al.’s (2016) findings more strongly suggest that a period of >1 month may remediate some of the sensory deficits related to AYA cannabis use (128, 130).

##### 3.3.3.2 Summary of EEG findings

The included EEG studies found that AYA cannabis use was associated with brain activity changes linked to sensory gating, executive function, and memory. Due to the small number of these studies overall as well as studies assessing sex/gender or abstinence, more research on the brain effects of AYA cannabis use using EEG is warranted. However, when comparing the included studies which assessed abstinence to each other, their results suggest that abstinence may reverse AYA cannabis use-related changes to brain activity detected by EEG.

#### 3.3.4 PET

Of all studies included in the current review, only 2 used PET. Cox et al. (2020) reported an interaction between adolescent cannabis use and level of externalizing traits, such that participants who scored highly on their measure of externalizing traits and used cannabis regularly had a significantly decreased availability of glutamate receptors in their orbitofrontal cortex, striatum, and insula (133). Sevy et al. (2008) found that male adolescents who were former cannabis users had decreased glutamate metabolism in the putamen, right cerebellum, precuneus, right orbitofrontal cortex, and the right medial posterior parietal cortex (134).

##### 3.3.4.1 Abstinence and PET

Sevy et al.’s (2008) finding of deficient glutamate metabolism in cannabis users was found in participants who were abstinent for at least one month [*M* = 15 weeks; (134)].

##### 3.3.4.2 Summary of PET findings

Due to only 2 studies in the current review using PET to assess the brains of AYA cannabis users, no sweeping conclusions can be made generally or regarding sex/gender and abstinence.

## 4 Discussion

The purpose of this scoping review was to summarize the existing evidence regarding the effect that cannabis use during AYA (ages 14-25) may have on the structure, function, and metabolic function of the brain. Our summary of existing literature suggests that AYA cannabis use is associated with changes in cortical thickness, brain volumes, neuronal white matter microstructure, metabolite concentrations, task-based and resting-state functional brain activity, and neurotransmitter metabolism/receptor density. However, these results must be considered in light of the limitations of a scoping review, namely being the absence of evidence quality assessments and meta-analyses. These findings should also be considered alongside the goal of the current review, which was not to provide an answer to the question of AYA cannabis use’s effect on the brain, but to collate and summarize relevant research.

Regarding the effects of abstinence from cannabis use on the brain, we found a limited number of conflicting findings. The majority of included studies which assessed abstinent AYA cannabis users found that cannabis-related changes persisted over time (45, 56, 64, 68, 84, 88, 94, 97, 128, 134), with one study finding that changes in the thickness of the layers of the hippocampus persisted for decades (37). The remaining studies instead found that abstinent AYA cannabis users did not differ from non-users on their particular neuroimaging measure (63, 111, 127, 130). However, only one of these latter studies assessed the participants pre- and post-abstinence (127), limiting the usefulness of these results. Overall, these findings suggest that abstinence does not necessarily reverse cannabis-related brain changes when use is initiated in adolescence or emerging adulthood, which is concerning for this age group considering the brain development that occurs during this time (7).

Regarding sex and/or gender differences related to the brain effects of cannabis in the included studies, only 17/99 explicitly assessed how sex/gender impacted their results. Of these 17, only 5 found a significant sex or gender difference (54, 55, 77, 88, 98). McQueeny et al. (2011), Maple (2016), and Sullivan et al. (2022) assessed gender, while Muetzel et al. (2013) and Dager et al. (2018) assessed sex. In addition to these significant findings, Medina et al. (2009) found a trend-level effect of gender on AYA cannabis use-related brain volume changes (63). These findings are salient enough to elicit AYA sex/gender considerations as well as concern over how rare the practice of assessing sex/gender differences in conjunction with cannabis use in neuroimaging studies is. The potential interaction between sex/gender and cannabis brain effects with this AYA cohort is important in our further biological understanding of these interactions, as well as any sex/gender specific knowledge translation activity. It is thus important that future neuroimaging research investigating the effects of AYA cannabis use on the brain incorporates sex/gender analyses into their designs.

The current review has multiple strengths: we had our searches developed with the help of evidence synthesis experts and topic experts, we had two independent reviewers screen and extract data from all included studies, and we searched through multiple databases and preprint servers. However, the aforementioned results need to be considered in light of the limitations of scoping review methodology (34), We did not include an assessment of bias or assessment of the quality of the included studies. While a systematic review and meta-analysis could evaluate the quality of the evidence, a scoping review aggregates the results to a more easily actionable state. Systematic reviews and meta-analyses necessitate a more specific question and more restrictive inclusion criteria which would not allow us to properly assess the range of evidence that exists regarding the effect of adolescent/young adult cannabis use on the brain (27). In short, although a systematic review and meta-analysis would provide more rigor than a scoping review, it would require far more resources and limit the amount of evidence available for review. Another limitation of our review is the fact that it included mostly cross-sectional studies, which does not allow the establishment of temporal precedence, meaning that it is just as likely that brain abnormalities reported in this review could have existed prior to- or even led to AYA cannabis use. Finally, with this scoping review, cannabis use patterns, as well as preferred cannabis product used, were not a primary focus. Further research should consider these and other variables to inform the literature.

There are many potential implications of this review, including at the policy level. A prudent review and assessment of age of access to cannabis products, in those jurisdictions where cannabis is legalized, is warranted. Governments and local authorities should also invest in continued age-specific education around the potential brain effects resulting from AYA cannabis use and AYA specific interventions/harm reduction techniques. Finally, research needs to not only continue in this area, but it needs to do so with more consistency and rigor.

## 5 Conclusion

Here we show that there is evidence that cannabis use during AYA is associated with changes to brain structure and function, that these effects can still be seen after varying periods of abstinence, and that the sex/gender of the user might influence the relationship between AYA cannabis use and brain changes. Even though it is not the goal of the current review, it is worth noting that coming to a strong conclusion about the relationship between AYA cannabis use and brain changes would be exceedingly difficult due to the heterogeneity of the existing literature. Despite this, there are some consistent signals that are coming out of the literature showing, on balance, a negative impact on brain structure and function with cannabis use in this age group. What is difficult to comment on at this time, is the potential varying effects of amounts of cannabis used and potency of product on these brain imaging variables, and effects of longer period of abstinence. With few studies examining sex/gender, little can be said on these specific effects as well, so we recommend that future research meaningfully consider detailed cannabis use patterns/products used and sex/gender in their designs and analyses.

## Appendix I: database search terms

**CINAHL (EBSCO)**

(MH “Cannabis”) OR (MH “Cannabinoids”) OR (MH “Cannabidiol”) OR TI ((cannabis or marijuana or “thc” or pot or weed or “cbd” or tetrahydrocannabinol or cannabidiol or Cannabinoid*)) OR AB ((cannabis or marijuana or “thc” or pot or weed or “cbd” or tetrahydrocannabinol or cannabidiol or Cannabinoid*)).

AND

(MH “Adolescence”) OR (MH “Young Adult”) OR TI (adolescen* or “young adult*” or teen* or youth* or “Young person” OR “young people” OR “emerging adult*” or juvenile) OR AB (adolescen* or “young adult*” or teen* or youth* or “Young person” OR “young people” OR “emerging adult*” or juvenile).

AND

(MH “Neuroradiography”) OR (MH “Brain Cortical Thickness”) OR (MH “Dopaminergic Imaging”) OR (MH “Echoencephalography”) OR TI (“Neuroradiography” OR “Brain Cortical Thickness” OR “Dopaminergic Imaging” OR “Echoencephalography” OR brain or “white matter” OR “grey matter” OR “gray matter” OR “functional imaging” OR “structural imaging” OR spectroscop* OR “MRI” “Magnetic resonance imaging” OR”FMRI” OR “DTI” OR “Diffusion tensor imaging” OR MRS OR “Magnetic resonance spectroscopy” OR “prefrontal cortex” OR “pre-frontal cortex” OR “neural pathways” OR “connectivity” OR “neuroimaging” OR “fractional anisotropy” OR FA OR “resting state” OR DWI OR “diffusion weighted imaging”) OR AB (“Neuroradiography” OR “Brain Cortical Thickness” OR “Dopaminergic Imaging” OR “Echoencephalography” OR brain or “white matter” OR “grey matter” OR “gray matter” OR “functional imaging” OR “structural imaging” OR spectroscop* OR “MRI” “Magnetic resonance imaging” OR”FMRI” OR “DTI” OR “Diffusion tensor imaging” OR MRS OR “Magnetic resonance spectroscopy” OR “prefrontal cortex” OR “pre-frontal cortex” OR “neural pathways” OR “connectivity” OR “neuroimaging” OR “fractional anisotropy” OR FA OR “resting state” OR DWI OR “diffusion weighted imaging”).

**Ovid MEDLINE (NLM, Wolters Kluwer)**

Cannabis/OR (cannabis or cannabinoid* or cannabidiol or marijuana or “THC” or pot or weed or “CBD” or tetrahydrocannabinol).ti,ab.

AND

Adolescent/OR Young Adult/OR (adolescen* or “young adult*” or teen* or youth* or “Young person” or “young people” or “emerging adult*” or juvenile).ti,ab.

AND

positron emission tomography computed tomography/or single photon emission computed tomography computed tomography/or neuroimaging/or brain cortical thickness/or diffusion tensor imaging/or functional neuroimaging/or brain mapping/or connectome/or dopaminergic imaging/or exp magnetic resonance imaging/OR (“positron emission tomography” or “computed tomography” or “single photon emission computed tomography” or brain or “white matter” or “grey matter” or “gray matter” or “functional imaging” or “structural imaging” or spectroscop* or “MRI Magnetic resonance imaging” or “FMRI” or “DTI” or “diffusion tensor imaging” or “MRS” or “Magnetic resonance spectroscopy” or “prefrontal cortex” or “pre-frontal cortex” or “neural pathways” or “connectivity” or “neuroimaging” or “fractional anisotropy” or “FA” or “resting state” or “DWI” or “diffusion weighted imaging”).ti,ab.

**PsycINFO (American Psychological Association).**

cannabis/or cannabinoids/or marijuana/OR “cannabis use”/OR “cannabis use disorder”/OR (cannabis or cannabinoid* or cannabidiol or marijuana or “THC” or pot or weed or “CBD” or tetrahydrocannabinol).ti,ab.

AND

exp Late Adolescence/or exp Early Adolescence/OR emerging adulthood/OR (adolescen* or “young adult*” or teen* or youth* or “Young person” or “young people” or “emerging adult*” or juvenile).ti,ab.

AND

neuroimaging/or encephalography/or magnetic resonance imaging/or brain connectivity/or diffusion tensor imaging/OR functional magnetic resonance imaging/OR white matter/OR gray matter/OR spectroscopy/OR (brain or “white matter” or “grey matter” or “gray matter” or “functional imaging” or “structural imaging” or spectroscop* or “MRI Magnetic resonance imaging” or “FMRI” or “DTI” or “Diffusion tensor imaging” or “MRS” or “Magnetic resonance spectroscopy” or “prefrontal cortex” or “pre-frontal cortex” or “neural pathways” or “connectivity” or “neuroimaging” or “fractional anisotropy” or “FA” or “resting state” or “DWI” or “diffusion weighted imaging” or encephalography).ti,ab.

**Embase (Elsevier)**

cannabis/or cannabinoid/OR cannabis addiction/OR cannabis smoking/or “cannabis use”/OR (cannabis or cannabinoid* or cannabidiol or marijuana or “THC” or pot or weed or “CBD” or tetrahydrocannabinol).ti,ab.

AND

adolescent/or juvenile/OR young adult/OR (adolescen* or “young adult*” or teen* or youth* or “Young person” or “young people” or “emerging adult*” or juvenile).ti,ab.

AND

neuroimaging/OR functional neuroimaging/OR nuclear magnetic resonance spectroscopy/OR diffusion tensor imaging/OR diffusion weighted imaging/OR white matter/OR gray matter/OR (brain or “white matter” or “grey matter” or “gray matter” or “functional imaging” or “structural imaging” or spectroscop* or “MRI Magnetic resonance imaging” or “FMRI” or “DTI” or “Diffusion tensor imaging” or “MRS” or “Magnetic resonance spectroscopy” or “prefrontal cortex” or “pre-frontal cortex” or “neural pathways” or “connectivity” or “neuroimaging” or “fractional anisotropy” or “FA” or “resting state” or “DWI” or “diffusion weighted imaging”).ti,ab.

**MedRxiv and Google Scholar.**

cannabis OR marijuana.

AND

adolescen* OR teen* OR youth* OR young.

AND

neuroimaging OR MRI OR MRS OR brain.
